# Predictability of parental ultraviolet-B environment shapes the growth strategies of clonal *Glechoma longituba*

**DOI:** 10.3389/fpls.2022.949752

**Published:** 2022-08-04

**Authors:** Yuehan Guo, Jiaxin Quan, Xubo Wang, Zhixing Zhang, Xiao Liu, Ruichang Zhang, Ming Yue

**Affiliations:** Key Laboratory of Resource Biology and Biotechnology in Western China, Ministry of Education, Northwest University, Xi’an, China

**Keywords:** environmental predictability, phenotypic plasticity, clonal plants, UV-B radiation, maternal effects, transgenerational effect, within-generational plasticity

## Abstract

Although there is an increasing debate about ecological consequences of environmental predictability for plant phenotype and fitness, the effect of predictability of parental environments on the offspring is still indefinite. To clarify the role of environmental predictability in maternal effects and the growth strategy of clonal offspring, a greenhouse experiment was conducted with *Glechoma longituba*. The parental ramets were arranged in three ultraviolet-B (UV-B) conditions, representing two predictable environments (regular and enhanced UV-B) and an unpredictable environment (random UV-B), respectively. The offspring environments were the same as their parent or not (without UV-B). At the end of experiment, the growth parameters of offspring were analyzed. The results showed that maternal effects and offspring growth were regulated by environmental predictability. Offspring of unpredictable environmental parents invested more resources in improving defense components rather than in rapid growth. Although offspring of predictable parents combined two processes of defense and growth, there were still some differences in the strategies between the two offspring, and the offspring of regular parent increased the biomass allocation to roots (0.069 g of control vs. 0.092 g of regular), but that of enhanced parent changed the resource allocation of nitrogen in roots and phosphorus in blade. Moreover, when UV-B environments of parent and offspring were matched, it seemed that maternal effects were not adaptive, while the growth inhibition in the predictable environment was weaker than that in unpredictable environment. In the predictable environment, the recovered R/S and the increased defense substances (flavonoid and anthocyanin) contributed to improving offspring fitness. In addition, when UV-B environments of parent and offspring were mismatched, offspring growth was restored or improved to some extent. The offspring performance in mismatched environments was controlled by both transgenerational effect and within-generational plasticity. In summary, the maternal effects affected growth strategies of offspring, and the differences of strategies depended on the predictability of parental UV-B environments, the clone improved chemical defense to cope with unpredictable environments, while the growth and defense could be balanced in predictable environments. The anticipatory maternal effects were likely to improve the UV-B resistance.

## Introduction

Variability is an intrinsic character of natural environment, and the magnitude and frequency of environmental variability are predicted to increase due to anthropogenic environmental change (Kochanek et al., 2011; Smale et al., 2019; Bintanja et al., 2020). Although plants can perceive the changes in environment and adapt to new conditions by adjusting their phenotype (Bornette and Puijalon, 2011; Li et al., 2021), variability and predictability of environment complicated the growth of plants. Plant phenotyping is a consequence of the interaction between genotype and environment (Walter et al., 2015; Pratap et al., 2019). Phenotypic plasticity is the main mechanism for plants to respond to changing environments and fast-changing climates (Sultan, 2001; Matesanz and Milla, 2017; Khodadadi et al., 2021). Phenotypes of offspring are determined not only by the genetic inheritance of causative alleles, but also by non-genetic influences of their environments and the environments experienced by parental generations (Auge et al., 2017a). Whether the environments of parent and offspring can be a cue to accurately predict the selective environment experienced by offspring, which will have different effects on the offspring phenotypes. In addition, the accuracy of this prediction will bring the offspring phenotype nearer to or further away from the optimum phenotype in the new environment (Sultan et al., 2009; Herman and Sultan, 2011; Auge et al., 2017a).

Maternal effects occur when the environment experienced by the mother influences the offspring phenotype over and above the direct effect of transmitted genes (Marshall and Uller, 2007). Despite the importance of maternal effects has been confirmed by many studies (Galloway and Etterson, 2007; Marshall and Uller, 2007; Auge et al., 2017b; Lyu et al., 2017; Donelson et al., 2018; Zettlemoyer and Lau, 2021; Zhou et al., 2021), the adaptive significance of maternal effects is often controversial. Some studies have found that adaptive maternal effects are widespread, allowing offspring to cope with rapidly changing environments or even increase fitness (Yin et al., 2019; Donelan et al., 2020). However, others showed weak evidence for adaptive maternal effects, and maternal effects may even reduce offspring fitness in these studies (Marshall and Uller, 2007; Uller et al., 2013). The contrasting results may be caused by the variability of the environment experienced by the parents.

Clonal plants are dominant species in many habitat types (Song et al., 2001). Compared with non-clonal plants, clonal offspring are thought to have a stronger ability to store the environmental information of parent for their asexual reproduction properties (Thellier and Lüttge, 2013; Vít et al., 2016; Richards et al., 2017). Although there have been some reports about the effect of parental environment on adaptability of clonal plants (Herman and Sultan, 2016; González et al., 2018; Baker et al., 2019; Dong et al., 2019), few studies consider the influence of environmental predictability of parent on the growth strategies of clonal offspring.

As an intrinsic part of the solar spectrum, ultraviolet-B (UV-B, 280–315 nm) light has many effects on the growth and development of plants (Liu et al., 2015; Quan et al., 2021). The effect of UV-B radiation on plants is comprehensive, and low-intensity UV-B radiation acts as a specific regulator for plants, while high-intensity UV-B radiation plays a negative effect on the growth and development of plants (Willing et al., 2016; Yin and Ulm, 2017). Low-intensity UV-B radiation regulates plant photomorphogenesis and thermomorphogenesis *via* UVR8 photoreceptor (Parihar et al., 2015; Hayes et al., 2017). Nevertheless, relatively high intensities of UV-B irradiation can damage macromolecules, inhibit photosynthesis, depress leaf expansion, reduce plant height, decrease biomass, and consequently affect plant growth, development, and morphology (Hectors et al., 2007; Berli et al., 2010; Liu et al., 2011). Therefore, variation of UV-B intensity in nature, such as the light environment under the forest, complicates the growth of plants (Bais et al., 2015; Robson et al., 2015).

In this study, parental ramets of clonal plant *Glechoma longituba* were assigned to three UV-B conditions, which represented two predictable environments (regular and enhanced UV-B) and an unpredictable environment (random UV-B), respectively. The offspring ramets were divided into two groups: One grew in the same UV-B environment as their parents, and the other was in an ambient light condition. The growth parameters of offspring were explored to evaluate (1) the difference of maternal effects caused by parental UV-B environment on the phenotypes and growth strategies of clonal offspring; (2) whether maternal effects help to improve the offspring adaptability when parental–offspring environments matched; and (3) the effect of offspring environments on its performance. We hypothesized that (1) both maternal effects and offspring environments significantly affect the phenotypes and growth strategies of clonal offspring, and the difference of growth strategies depends on environmental predictability, and (2) maternal effects contributed to improve offspring adaptation when the parental and offspring environments were identical.

## Materials and methods

### Plant material and propagation

*Glechoma longituba* (Nakai) Kuprian, a perennial clonal plant of the Lamiaceae family, was used in this experiment. This species produces long stolons with ramets on its nodes and is commonly employed in clonal plant research due to its high phenotypic plasticity (Liu et al., 2015). The *G. longituba* in our experiment was collected from Jiwozi in the Qinling Mountains, Shaanxi, China. The plant materials were collected from a genet to ensure the uniform of the genotypes and then were vegetatively propagated for at least 4 months in a greenhouse at Northwest University in Xi’an (34.3°N, 108.9°E; altitude 397 m a.s.l.) to reduce the impact of the previous environment through acclimatization.

The experiment was conducted in our greenhouse from May to August 2021. A total of 56 healthy ramets of similar size were selected as parental ramets and transplanted individually to plastic pots (7 cm length × 7 cm width × 7.8 cm depth) filled with nutrient soil (peat soil, perlite, vermiculite, and coconut bran). During the experiment, the culture conditions of the greenhouse were a 24/20°C day/night temperature cycle and a 13/11-h light/dark cycle, the mean irradiance was 150 μmol⋅m^–2^⋅s^–1^, and the relative humidity was maintained at 40%. Ramets were watered every 3 days to prevent water stress.

### Experimental design

The experiment was carried out in two stages: The first was the growth stage of parental ramets, and the second was that of offspring ramets. In the first stage, 56 ramets of similar size were selected as parents and divided into four groups randomly. One group was used as a control treatment, which grew in the ambient environment of the greenhouse. There was only a very low intensity of UV-B radiation (0.2 μW⋅cm^–2^) in the greenhouse. The other three groups were treated with UV-B radiations in different ways: random, regular, and enhanced UV-B radiation, which represented two predictable environments (regular and enhanced UV-B) and an unpredictable environment (random UV-B), respectively. This unpredictable random UV-B radiation was presented with the random treatment of UV-B intensity and frequency. The detail of UV-B treatments was described in the section of “Ultraviolet-B radiation treatments.” The control group was designed with eight replicates, and the UV-B treatment groups were designed with 16 replicates.

After 27 days of growth, parental ramets had grown about eight offspring ramets. According to our previous study, epigenetic variation caused by maternal UV-B environment can maintain in the third offspring ramets (Zhang et al., 2021). Therefore, the third offspring ramet was removed and replanted as the material of the second stage experiment. To avoid confusion, we described this third offspring ramet as the initial ramet of the second stage. In the second stage, half of the initial ramets in each treatment were placed in the same environment as their parents, and the other half were in the ambient environment as control groups. The offspring also grew for 27 days, the same time as their parents in the first stage.

**FIGURE 1:**
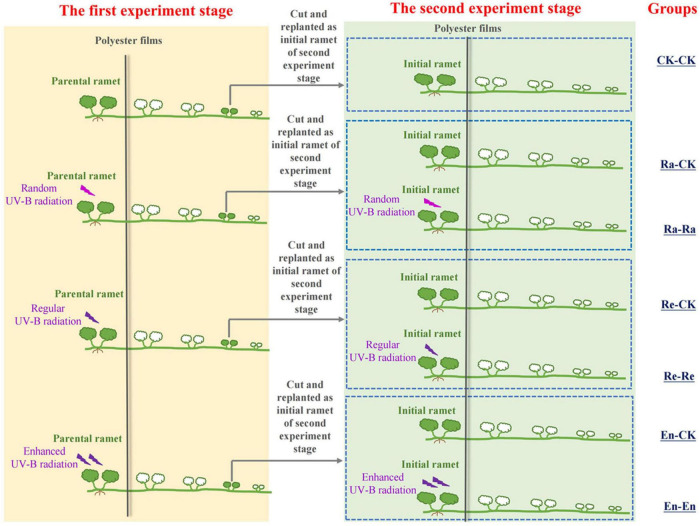
Schematic of the experiment. The experimental design consisted of seven treatments according to the condition of the offspring ramet in the second stage.

Therefore, there were seven treatments in the second stage experiment: CK-CK, Ra-Ra, Ra-CK, Re-Re, Re-CK, En-En, and En-CK. The details are described in Figure 1 and Table 1. In this experiment, there were three matched parental–offspring environments: Both parental ramets and initial ramets were grown under UV-B environments (Ra-Ra, Re-Re, and En-En). Meanwhile, three types of mismatched parental–offspring environments were selected: Parental ramets were grown under the UV-B conditions, and initial ramets were transplanted in an ambient condition (Ra-CK, Re-CK, and En-CK). In the whole process of the experiment, for the UV-B radiation treatments, only the parental ramets in the first stage experiment and the initial ramets of the second stage experiment were irradiated with UV-B, and other newborn ramets grew in an ambient greenhouse environment without additional UV-B treatment.

**TABLE 1:** Different treatments conducted in the study.

Treatments	Interpretation
CK-CK	Control group, all the ramets grew in the ambient environment of greenhouse during experiment, whatever that were in the first or second stage.
Ra-Ra	Only the parent in the first stage and the initial ramet in the second stage were treated with UV-B radiation randomly. Other ramets grew in the ambient environment of greenhouse. UV-B radiation (5 μW⋅cm^–2^) was controlled by turning off the UV-B lamps 4–6 times for 8 h radiation (9:00 a.m.–17:00 p.m.) per day, and the duration of interruption was 5–20 min each time.
Ra-CK	Except for the parental ramets of the first stage were treated with random UV-B radiation, other ramets all grew in the ambient environment of greenhouse.
Re-Re	Only the parent in the first stage and the initial ramet in the second stage were treated with UV-B radiation regularly, that means UV-B radiation (5 μW⋅cm^–2^) lasted 8 h (from 9:00 a.m. to 17:00 p.m.) per day. Other ramets all grew in the ambient environment of greenhouse.
Re-CK	Except for the parent of the first stage were treated with regular UV-B radiation, other ramets all grew in ambient light of greenhouse.
En-En	Only the parent in the first stage and the initial ramet in the second stage were treated with enhanced UV-B radiation. That means, the intensity of UV-B radiation was enhanced every 3 days (5, 6, 7, 8, 8.5, 9, 9.5, 10, and 10.5 μW⋅cm^–2^, respectively) and the radiation duration was 8 h (from 9:00 a.m. to 17:00 p.m.) per day. Other ramets all grew in ambient light of greenhouse.
En-CK	Except for the parent of the first stage were treated with enhanced UV-B radiation, other ramets all grew in ambient light of greenhouse.

### Ultraviolet-B radiation treatments

There were three UV-B radiation treatments involved in our experiment (random radiation, regular radiation, and enhanced radiation). In all treatments, the UV-B lamps were suspended above the plants, and the intensity of UV-B radiation was adjusted by modifying the distance between the lamps and the canopy. The differences among treatments were the intensity and frequency of UV-B radiation. The random UV-B radiation was controlled by turning off the lamp 4–6 times during 9:00 a.m.–17:00 p.m. per day randomly. To ensure the randomicity of random UV-B radiation, the frequency and duration of UV-B radiation were designed with the “dplyr” package of R software (RStudio, Auckland, New Zealand). The regular UV-B radiation treatment was conducted with UV-B radiation regularly, which means UV-B radiation (5 μW⋅cm^–2^) lasted 8 h (from 9:00 a.m. to 17:00 p.m.) per day. The plants of enhanced UV-B treatment were exposed to the increased UV-B radiation, the intensity of UV-B was improved every three days (5, 6, 7, 8, 8.5, 9, 9.5, 10, and 10.5 μW⋅cm^–2^, respectively), and the radiation duration was 8 h (from 9:00 a.m. to 17:00 p.m.) per day.

Supplementary UV-B radiation was artificially supplied by square-wave UV-B fluorescent lamps (36 W, Beijing Lighting Research Institute, Beijing, China) according to the method of Zhang et al. (2021). The maximum output wavelength of these lamps was 313 nm. During the experiment, these lamps were wrapped with either 0.13-mm cellulose acetate film (Grafix Plastics, Cleveland, OH, transmission down to 290 nm) for the supplemental UV-B radiation groups or with 0.13-mm polyester plastic film (absorbs radiation below 320 nm, Grafix Plastics) for the control group. Thus, the spectral difference between the control and UV-B groups is the presence or absence of UV-B. The cellulose acetate and polyester plastic films were replaced every 5 days. Furthermore, during the experiment, to avoid the interference from maternal UV-B radiation on the newborn ramets, the transparent polyester film (0.3 mm, Dongguan Linuo Plastic Insulation Material Co. Ltd., China) was placed vertically on both sides of the parental ramets in the first stage and the initial ramets of the second stage separately to ensure that the bottom of the films did not affect the growth of the newborn ramets. The intensity of UV-B radiation of different treatments was measured with an UV radiometer (Handy, Beijing, China).

### Measurement of parameters

At the beginning of the experiment, each treatment was repeated eight times; however, during the experiment, some plants died. At the end of the experiment, the ramets with healthy growth state were selected for parameter measurement and statistical analysis, and each treatment at least had three replicates. The initial ramets and whole clones of the second stage were harvested carefully according to the needs of measurement and analysis. The following growth parameters of initial ramet were measured: biomass of leaf, blade, petiole, node and roots, petiole length, blade area, flavonoid and anthocyanin content of blades, total carbon (TC) and total nitrogen (TN) of roots, total phosphorus (TP), nitrate-nitrogen, ammonium nitrogen, and organic carbon (OC) of blade. Moreover, the clones were harvested, and length of the longest stolon, number of ramets, branching intensity, the biomass of leaf, stolon, and roots were measured.

### Growth parameters

The leaf, blade, petiole, and roots of ramets, and the leaf, stolon, and roots of clones were collected for biomass measurement. These samples were dried at 75°C for 48 h to a constant weight, and biomass was measured immediately with an electronic balance (Sartorius BT25S, Beijing, China). Root–shoot ratio (R/S) was calculated by the ratio of root biomass to aboveground biomass; aboveground biomass of ramets was calculated as the sum of the biomass of blade, petiole, and node, while aboveground biomass of clone was calculated as the sum of the biomass of stolon and leaf; total biomass was obtained by summing aboveground and root biomass.

The fresh blades and petioles of the initial ramets and the longest stolons of clones were collected for the measurement of specific leaf area (SLA), specific petiole length (SPL), and specific stolon length (SSL) according to the method of Liu et al. (2015) and Zhu et al. (2018). Fresh blades were scanned with a scanner (Perfection V19, EPSON, China); then, blade area was calculated with Motic software (Motic Images Plus 2.0. Ink, Motic, China). Petiole length and stolon length were measured with a vernier caliper. SLA was calculated as the ratio of blade area to blade biomass. SPL was calculated as the ratio of petiole length to petiole biomass, and the ratio of stolon length to stolon biomass was calculated as SSL.

### Ultraviolet-B absorbing compound concentration

The UV-B absorbing compound content of fresh blades was measured as described by Liu et al. (2015). Blade disks were soaked in 4-ml centrifuge tubes containing methanol, HCl, and distilled H_2_O (79:1:20 volume) for 48 h in darkness. The concentration of UV-B absorbing compounds, mainly flavonoids and anthocyanins, was estimated by measuring the absorbance at 300 and 530 nm with a multimode microplate reader (Infinite 200 PRO NanoQuant, TECAN, Switzerland). The absorbance was used as an index of the relative concentration of UV-B absorbing compounds.

### Resource allocation

After drying treatment, the collected blades and roots of the initial ramets in the second experiment stage were crushed with a high-flux tissue grinder (Scientz-48, Ningbo Xinzhi, China), and dried powdered samples were used for resource allocation analysis. The contents of TC and TN in the roots and the contents of OC, ammonium nitrogen, nitrate-nitrogen, and TP in the blades were determined. The contents of ammonium nitrogen, nitrate-nitrogen, and TP in blades were digested by H_2_SO_4_-H_2_O_2_ and then determined by continuous flow analyzer (SEAL AutoAnalyzer3), ultraviolet and visible spectrophotometry, and Mo-Sb antispetrophotography method. The OC content in blades was determined by potassium dichromate external heating method. The contents of TC and TN in roots were determined by a German element analyzer (vario MACRO cube). TN in blades was obtained from ammonium nitrogen plus nitrate-nitrogen. Then, the N:P in blades and the C:N in roots were calculated.

### Statistical analysis

Before the statistical analyses, data were checked for normality and homoscedasticity using Shapiro–Wilk and Levene’s tests, and Blom transformation (Zhang et al., 2015) was used to achieve normality for non-normal data in SPSS Statistics 24.0 software (IBM, United States). Then, considering the slight difference among the initial biomass of initial ramets, analysis of covariance (ANCOVA) was used to test the effects of parental environment and current environment of offspring on the growth characters (biomass, leaf parameters, defensive substances, and growth architecture) and resource allocation of offspring. Duncan’s test was chosen as the method of multiple comparisons to test the significance among different treatments, and the significance level was set at the *P* < 0.05 level. All ANCOVA analyses were performed with STATISTICA 6.0 software (StatSoft, Tulsa, OK, United States). In addition, the data of ramet number and branching intensity still did not accord with the normal distribution after Blom transformation, so they were analyzed by the Kruskal–Wallis test of non-parametric test to determine the effect of different environments on the growth architecture of whole clones. Analytical mapping was performed using Origin Pro 8.0 software (OriginLab, United States).

## Results

### The influence of parental environment on the growth of offspring

#### Mismatched parental–offspring environments

To study the maternal effect triggered by parental environment on offspring performance, the offspring growth in ambient environments was analyzed, and the parents of these offspring were exposed to different UV-B radiation (Tables 2, 3). Compared with the CK-CK, the total biomass of ramet decreased significantly in the Ra-CK (0.336 g of CK-CK vs. 0.292 g of Ra-CK; *P* < 0.05), which was caused by the overall decrease in aboveground and underground biomass. In addition, the decrease in aboveground biomass was mainly caused by the decrease in blades biomass. There was no significant change in R/S between the CK-CK and Ra-CK (0.249 of CK-CK vs. 0.244 of Ra-CK; *P* > 0.05). There were no significant differences in ramet biomass (total, aboveground, leaf, and petiole biomass) among the CK-CK, Re-CK, and En-CK treatments (*P* > 0.05), but the changing trend of root biomass was different, and the root biomass in Re-CK increased significantly (0.092 vs. 0.069 g of control; *P* < 0.05), which led to the increase in R/S (0.379 vs. 0.249 of CK-CK; *P* < 0.05). Although R/S also increased (0.321 of En-CK vs. 0.249 of CK-CK; *P* < 0.05), there was no significant difference in root biomass between the CK-CK and En-CK (*P* > 0.05), and the highest increase in R/S was displayed in Re-CK.

**TABLE 2:** Growth parameters of offspring in the ambient environment.

Traits	CK-CK	Ra-CK	Re-CK	En-CK
**Ramets biomass**				
Total (g)	0.336 ± 0.009 a	0.292 ± 0.010 b	0.341 ± 0.018 a	0.321 ± 0.013 ab
Aboveground (g)	0.266 ± 0.007 a	0.231 ± 0.007 b	0.250 ± 0.016 ab	0.244 ± 0.008 ab
Root (g)	0.069 ± 0.002 b	0.057 ± 0.002 c	0.092 ± 0.003 a	0.074 ± 0.004 b
R/S	0.249 ± 0.008 c	0.244 ± 0.008 c	0.379 ± 0.012 a	0.321 ± 0.010 b
Leaf (g)	0.257 ± 0.006 a	0.224 ± 0.007 b	0.241 ± 0.016 ab	0.239 ± 0.008 ab
Blade (g)	0.236 ± 0.006 a	0.204 ± 0.006 b	0.213 ± 0.014 b	0.219 ± 0.008 ab
Petiole (g)	0.021 ± 0.001 a	0.020 ± 0.001 a	0.021 ± 0.001 a	0.020 ± 0.001 a
**Leaf parameters**				
Petiole length (cm)	3.906 ± 0.093 b	4.050 ± 0.107 ab	4.246 ± 0.094 a	4.125 ± 0.088 ab
SPL (cm/g)	371.739 ± 8.789 b	411.962 ± 9.300 a	411.783 ± 15.766 a	416.197 ± 17.491 a
Blade area (cm^2^)	58.237 ± 1.099 a	53.756 ± 1.758 a	60.185 ± 3.915 a	59.682 ± 3.440 a
SLA (cm^2^/g)	248.486 ± 5.853 b	265.085 ± 7.287 ab	276.506 ± 8.191 a	279.906 ± 9.153 a
**Defensive substances**				
Flavonoid (OD_300_)	0.681 ± 0.038 c	1.680 ± 0.071 a	1.335 ± 0.038 b	1.205 ± 0.043 b
Anthocyanin (OD_530_)	0.030 ± 0.001 c	0.075 ± 0.003 a	0.045 ± 0.006 b	0.035 ± 0.005 bc
**Clone biomass**				
Total (g)	0.923 ± 0.027 b	0.861 ± 0.064 b	1.188 ± 0.026 a	1.124 ± 0.038 a
Aboveground (g)	0.856 ± 0.025 b	0.838 ± 0.049 b	1.097 ± 0.023 a	1.043 ± 0.029 a
Leaf (g)	0.591 ± 0.016 b	0.526 ± 0.038 b	0.727 ± 0.023 a	0.708 ± 0.018 a
Stolon (g)	0.244 ± 0.006 b	0.237 ± 0.021 b	0.345 ± 0.011 a	0.330 ± 0.016 a
Leaf biomass/total biomass	0.649 ± 0.008 a	0.653 ± 0.007 a	0.620 ± 0.008 b	0.636 ± 0.009 ab
Stolon biomass/total biomass	0.269 ± 0.006 b	0.270 ± 0.007 b	0.289 ± 0.006 a	0.291 ± 0.006 a
R/S	0.079 ± 0.003 b	0.076 ± 0.002 b	0.088 ± 0.003a	0.077 ± 0.004 b
**Growth architecture**				
Number of ramets	6.167 ± 0.146 a	6.333 ± 0.188 a	6.500 ± 0.151 a	6.583 ± 0.149 a
Branching intensity	2.500 ± 0.167 b	3.000 ± 0.211 ab	3.667 ± 0.142 a	3.125 ± 0.125 ab
Length of the longest stolon (cm)	47.536 ± 1.017 ab	45.025 ± 1.251 b	49.180 ± 0.963 ab	49.710 ± 1.530 a
SSL (cm/g)	378.115 ± 8.586 b	412.741 ± 14.801 a	343.379 ± 10.633 c	344.731 ± 10.067 c

Values with different letters were significantly different among four treatments, whereas the same letter indicates no significant difference among four treatments (*P* < 0.05). Data were mean ± SE (*n* ≥ 3). R/S, root–shoot ratio; SPL, specific petiole length; SLA, specific leaf area; SSL, specific stolon length.

**TABLE 3:** Resource allocation of initial ramet in the second experiment stage under ambient condition.

Organ	Parameters	CK-CK	Ra-CK	Re-CK	En-CK
Blades	Total phosphorus (g/kg)	8.04 ± 0.09 a	7.54 ± 0.19 ab	8.01 ± 0.17 a	7.24 ± 0.32 b
	Nitrate nitrogen (g/kg)	2.28 ± 0.06 b	2.69 ± 0.06 a	2.17 ± 0.05 b	2.09 ± 0.04 b
	Ammonium nitrogen (g/kg)	28.66 ± 0.20 a	28.61 ± 0.22 a	28.5 ± 0.66 a	27.81 ± 0.28 a
	Organic carbon (g/kg)	397.89 ± 6.50 a	399.64 ± 2.90 a	402.9 ± 2.87 a	406.81 ± 0.84 a
	Total nitrogen (g/kg)	30.94 ± 0.22 ab	31.3 ± 0.17 a	30.68 ± 0.61 ab	29.91 ± 0.29 b
	N:P	3.85 ± 0.07 a	4.16 ± 0.09 a	3.83 ± 0.03 a	4.15 ± 0.23 a
Roots	Total carbon (g/mg)	424.54 ± 2.04 b	439.54 ± 3.92 a	424.35 ± 1.61 b	427.78 ± 3.06 b
	Total nitrogen (g/kg)	22.73 ± 0.12 c	24.92 ± 0.21 a	22.61 ± 0.17 c	24.36 ± 0.08 b
	C:N	18.68 ± 0.02 a	17.64 ± 0.29 b	18.77 ± 0.21 a	17.56 ± 0.17 b

Values with different letters were significantly different among four treatments, whereas, the same letter indicates no significant difference among four treatments (*P* < 0.05). Data were mean ± SE (*n* ≥ 3). N:P, total nitrogen/total phosphorus (blades); C:N, total carbon/total nitrogen (roots).

There was no significant difference in petiole length, blade area, and SLA between the CK-CK and Ra-CK (*P* > 0.05), but the petiole became slender in the Ra-CK (411.962 cm/g of SPL vs. 371.739 cm/g of SPL in CK-CK; *P* < 0.05). Compared with CK-CK, the petiole in Re-CK and En-CK also became thin, and SLA was increased, but no difference was found in blade area. Furthermore, the petiole length increased only in the Re-CK by comparing with the CK-CK (4.246 vs. 3.906 cm of CK-CK; *P* < 0.05).

Concerning the content of defensive substances, the flavonoid and anthocyanin values of offspring in the CK-CK were 0.681 and 0.030. Compared with the CK-CK treatment, flavonoids were increased significantly in Ra-CK, Re-CK, and En-CK groups, while anthocyanins were increased in Ra-CK and Re-CK, but no difference was found in En-CK. The maximum value of flavonoid and anthocyanin both appeared in the Ra-CK (1.680 of flavonoid and 0.075 of anthocyanin).

There was no significant difference in clone biomass and biomass allocation between the CK-CK and Ra-CK (*P* > 0.05). The biomass of all parts of clone (biomass of stolon, leaf, and aboveground, and total biomass) and stolon biomass allocation (stolon biomass/total biomass) increased in the Re-CK and En-CK. But the leaf biomass allocation in the Re-CK group (leaf biomass/total biomass) decreased (0.620 vs. 0.649 of CK-CK; *P* < 0.05) and R/S increased (0.088 vs. 0.079 of CK-CK; *P* < 0.05). These two parameters had no differences between the CK-CK and En-CK (Table 2).

No significant difference in the number of ramets was found among the four treatments (*P* > 0.05). There were also no significant differences in branching intensity among the CK-CK, Ra-CK, and En-CK treatments. The increase in branching intensity was only observed in the Re-CK. The clone in the En-CK had longer stolon lengths (49.710 cm) by comparing with the Ra-CK (45.025 cm). Compared with the CK-CK (378.115 cm/g), the SSL increased in Ra-CK (412.741 cm/g) and decreased in both Re-CK (343.379 cm/g) and En-CK (344.731 cm/g). The longest stolon of the offspring in the Ra-CK became slender, but it became thicker in the Re-CK and En-CK (Table 2).

The value of nitrate-nitrogen of blade, TC, and TN of roots in Ra-CK was the highest in all groups. The TN level of roots increased in En-CK (24.36 g/kg vs. 22.73 g/kg of CK-CK; *P* < 0.05), but C:N in roots of En-CK was decreased (17.56 vs. 18.68 of CK-CK; *P* < 0.05). These parameters of resource allocation in Re-CK all did not display significant differences with CK-CK (Table 3).

#### Matched parental–offspring environments

In order to clarify whether maternal effects helped to improve the offspring adaptability when parental–offspring environments matched, the growth of offspring, which grew in the same UV-B environments as their parent, was compared (Tables 4, 5). The changing trends of initial ramet biomass were similar, showing a significant decrease in total, aboveground, root, leaf, blade, and petiole biomass. The minimum value of root biomass and petiole biomass both appeared in the Ra-Ra (0.022 g of root and 0.010 g of petiole). The R/S decreased significantly in the Ra-Ra (0.182 vs. 0.249 of CK-CK; *P* < 0.05). Compared with CK-CK, there was no significant difference in R/S in the Re-Re and En-En, but a larger increase in R/S was presented in En-En (0.227 of Re-Re vs. 0.273 of En-En; *P* < 0.05).

**TABLE 4:** Growth parameters of offspring in the different ultraviolet-B (UV-B) environments.

Traits	CK-CK	Ra-Ra	Re-Re	En-En
**Ramets biomass**				
Total (g)	0.336 ± 0.009 a	0.143 ± 0.011 c	0.203 ± 0.010 b	0.164 ± 0.012 bc
Aboveground (g)	0.266 ± 0.007 a	0.121 ± 0.009 c	0.169 ± 0.007 b	0.127 ± 0.008 c
Roots (g)	0.069 ± 0.002 a	0.022 ± 0.001 c	0.033 ± 0.003 b	0.033 ± 0.004 b
R/S	0.249 ± 0.008 ab	0.182 ± 0.012 c	0.227 ± 0.015 b	0.273 ± 0.011 a
Leaf (g)	0.257 ± 0.006 a	0.114 ± 0.009 c	0.163 ± 0.007 b	0.122 ± 0.009 c
Blade (g)	0.236 ± 0.006 a	0.104 ± 0.008 c	0.146 ± 0.008 b	0.112 ± 0.013 c
Petiole (g)	0.021 ± 0.001 a	0.010 ± 0.001 c	0.013 ± 0.001 b	0.012 ± 0.001 b
**Leaf parameters**				
Petiole length (cm)	3.906 ± 0.093 a	2.736 ± 0.112 c	3.161 ± 0.094 b	3.168 ± 0.089 b
SPL (cm/g)	371.739 ± 8.789 d	592.877 ± 24.937 a	483.448 ± 15.364 c	531.260 ± 25.509 b
Blade area (cm^2^)	58.237 ± 1.099 a	35.836 ± 1.502 bc	42.894 ± 2.237 b	31.189 ± 2.109 c
SLA (cm^2^/g)	248.486 ± 5.853 c	328.156 ± 7.964 a	293.605 ± 6.700 b	304.989 ± 8.320 b
**Defensive substances**				
Flavonoid (OD_300_)	0.681 ± 0.038 b	1.781 ± 0.075 a	1.930 ± 0.075 a	1.860 ± 0.074 a
Anthocyanin (OD_530_)	0.030 ± 0.001 c	0.023 ± 0.004 c	0.075 ± 0.003 a	0.049 ± 0.007 b
**Clone biomass**				
Total (g)	0.923 ± 0.027 a	0.399 ± 0.016 b	0.461 ± 0.025 b	0.512 ± 0.052 b
Aboveground (g)	0.856 ± 0.025 a	0.384 ± 0.017 b	0.442 ± 0.032 b	0.484 ± 0.048 b
Leaf (g)	0.591 ± 0.016 a	0.295 ± 0.010 c	0.340 ± 0.018 b	0.360 ± 0.032 b
Stolon (g)	0.244 ± 0.006 a	0.081 ± 0.005 c	0.112 ± 0.008 b	0.116 ± 0.013 b
Leaf biomass/total biomass	0.649 ± 0.008 c	0.736 ± 0.014 a	0.697 ± 0.011 b	0.688 ± 0.006 b
Stolon biomass/total biomass	0.269 ± 0.006 a	0.200 ± 0.006 c	0.223 ± 0.009 b	0.232 ± 0.006 b
R/S	0.079 ± 0.003 a	0.058 ± 0.002 b	0.078 ± 0.002 a	0.076 ± 0.002 a
**Growth architecture**				
Number of ramets	6.167 ± 0.146 a	5.444 ± 0.176 a	6.000 ± 0.289 a	6.000 ± 0.191 a
Branching intensity	2.500 ± 0.167 ab	2.000 ± 0.000 b	3.000 ± 0.236 a	3.286 ± 0.184 a
Length of the longest stolon (cm)	47.536 ± 1.017 a	29.957 ± 1.008 bc	28.700 ± 0.836 c	34.620 ± 1.649 b
SSL (cm/g)	378.115 ± 8.586 c	591.822 ± 38.130 a	523.747 ± 29.775 ab	483.232 ± 22.129 b

Values with different letters were significantly different among four treatments, whereas the same letter indicates no significant difference among four treatments (*P* < 0.05). Data were mean ± SE (*n* ≥ 3). R/S, root–shoot ratio; SPL, specific petiole length; SLA, specific leaf area; SSL, specific stolon length.

**TABLE 5:** Resource allocation of initial ramet in the second experiment stage under the different ultraviolet-B (UV-B) conditions.

Organ	Parameters	CK-CK	Ra-Ra	Re-Re	En-En
Blades	Total phosphorus (TP) (g/kg)	8.04 ± 0.09 a	3.99 ± 0.09 c	4.58 ± 0.15 b	4.79 ± 0.13 b
	Nitrate nitrogen (g/kg)	2.28 ± 0.06 c	3.21 ± 0.05 a	2.63 ± 0.06 b	2.55 ± 0.10 b
	Ammonium nitrogen (g/kg)	28.66 ± 0.20 a	23.47 ± 0.15 b	22.45 ± 0.11 c	23.75 ± 0.49 b
	Organic carbon (OC) (g/kg)	397.89 ± 6.50 c	427.21 ± 2.15 ab	424.20 ± 0.60 b	430.14 ± 1.56 a
	Total nitrogen (TN) (g/kg)	30.94 ± 0.22 a	26.68 ± 0.11 b	25.08 ± 0.16 c	26.31 ± 0.44 b
	N:P	3.85 ± 0.07 c	6.68 ± 0.17 a	5.49 ± 0.20 b	5.50 ± 0.06 b
Roots	Total carbon (TC) (g/mg)	424.54 ± 2.04 ab	412.78 ± 4.46 c	425.41 ± 1.36 a	414.23 ± 3.10 bc
	Total nitrogen (TN) (g/kg)	22.73 ± 0.11 a	19.55 ± 0.19 c	19.25 ± 0.21 c	21.39 ± 0.23 b
	C:N	18.68 ± 0.02 c	21.13 ± 0.41 b	22.10 ± 0.29 a	19.37 ± 0.07 c

Values with different letters were significantly different among four treatments, whereas, the same letter indicates no significant difference among four treatments (*P* < 0.05). Data were mean ± SE (*n* ≥ 3). N:P, total nitrogen/total phosphorus (blades); C:N, total carbon/total nitrogen (roots).

After the initial ramets experienced the matched parental–offspring UV-B radiation, the changing trend of leaf parameters was similar. The petiole became short and slender, the blade area decreased, and the SLA increased in the UV-B radiation groups by comparing with the CK-CK. In the Ra-Ra treatment, the petiole was the shortest and the slenderest, and the SLA was the largest. In addition, the SPL of the En-En was larger than that of the Re-Re (531.260 cm/g of En-En vs. 483.448 cm/g of Re-Re; *P* < 0.05), and the blade area of the En-En was smaller than that of the Re-Re (31.189 cm^2^ of En-En vs. 42.894 cm^2^ of Re-Re; *P* < 0.05). There was no significant difference in petiole length (3.161 vs. 3.168 cm; *P* > 0.05) and SLA (293.605 cm^2^/g vs. 304.989 cm^2^/g; *P* > 0.05) between Re-Re and En-En.

Flavonoids increased significantly in the radiation environments, and there was no significant difference among the three different UV-B treatments (0.681 of CK-CK vs. 1.781 of Ra-Ra, 1.930 of Re-Re, and 1.860 of En-En). There was no significant difference in anthocyanin content between the CK-CK and Ra-Ra (0.030 vs. 0.023; *P* > 0.05), but the content of anthocyanin in the Re-Re and En-En was increased significantly (0.075 of Re-Re; 0.049 of En-En) and the maximum value of anthocyanin was shown in the Re-Re.

The biomass of all parts of the clone (biomass of stolon, leaf, and aboveground, and total biomass) in the matched parental–offspring UV-B environments decreased significantly. The minimum value of the leaf (0.295 g) and stolon (0.081 g) biomass appeared in the Ra-Ra. In the UV-B treatments, the leaf biomass allocation increased significantly, the stolon biomass allocation decreased significantly, but the larger increase or decrease in biomass allocation was presented in Ra-Ra. There was no significant difference in clone biomass and biomass allocation between the Re-Re and En-En. The R/S decreased in the Ra-Ra group (0.058 vs. 0.079 of CK-CK; *P* < 0.05), but had no significant difference among the CK-CK, Re-Re, and En-En treatments (Table 4).

There was no difference among the different treatments in the number of ramets. The branching intensity in the Re-Re and En-En was more than that in the Ra-Ra. The longest stolon became short and slender after plants experienced UV-B radiation, and the maximum value of SSL was presented in the Ra-Ra group (591.822 cm/g). The longest stolon in the En-En (34.620 cm) was longer than that in the Re-Re (28.700 cm). The SSL indicated no significant difference between the Re-Re and En-En (523.747 cm/g vs. 483.232 cm/g; *P* > 0.05).

After UV-B radiation, the TP, ammonium nitrogen, and TN in blades decreased significantly, and nitrate-nitrogen, OC, and N:P increased significantly. Besides, the content of TN in root decreased significantly in UV-B treatments by comparing with the CK-CK. Compared with other treatments, the TP of blades in the Ra-Ra (3.99 g/kg) was the lowest, while nitrate-nitrogen (3.21 g/kg) and N:P (6.68) were the largest. There was no significant difference in TP, nitrate-nitrogen, and N:P between the Re-Re and En-En. The larger value in ammonium nitrogen (23.75 vs. 22.45 g/kg of Re-Re), OC (430.14 vs. 424.20 g/kg of Re-Re), TN of blade (26.31 vs. 25.08 g/kg of Re-Re), and TN of root (21.39 vs. 19.25 g/kg of Re-Re) was presented in En-En treatment, but the larger value in TC (425.41 vs. 414.23 g/mg of En-En) and C:N (22.10 vs. 19.37 of En-En) of root appeared in Re-Re (Table 5).

### The influence of offspring environment on their growth

**FIGURE 2:**
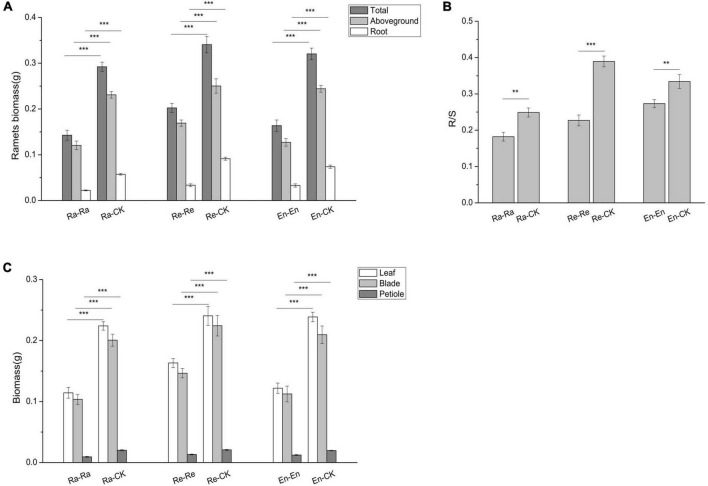
Biomass and its allocation of the initial ramet in the second experiment stage under different offspring conditions. **(A)** Ramets biomass; **(B)** root biomass/shoot biomass (R/S); **(C)** biomass of leaf, blade, and petiole. Different numbers of asterisks indicate significant differences of the same parameter between two groups, with “**” indicated *p* < 0.01, and “***” indicated *p* < 0.001. Error bars showed ± SE (*n* ≥ 3).

**FIGURE 3:**
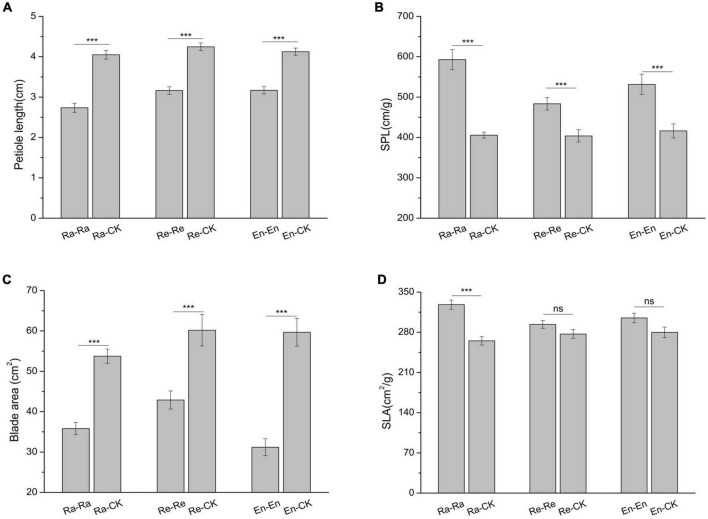
Leaf parameters of initial ramet in the second experiment stage under different offspring conditions. **(A)** Petiole length; **(B)** specific petiole length (SPL); **(C)** blade area; **(D)** specific leaf area (SLA). Different numbers of asterisks indicate significant differences of the same parameter between two groups, with “***” indicated *p* < 0.001, and “ns” indicated *p* > 0.05. Error bars showed ± SE (*n* ≥ 3).

**FIGURE 4:**
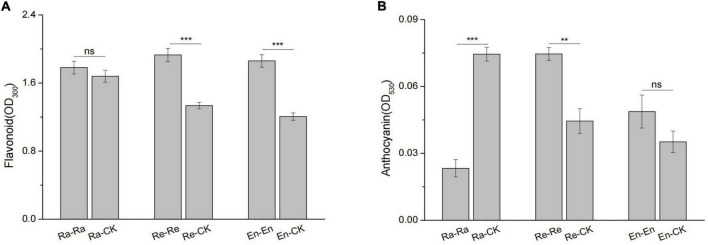
Defensive substances of initial ramet in the second experiment stage under different offspring conditions. **(A)** Flavonoid; **(B)** anthocyanin. Different numbers of asterisks indicate significant differences of the same parameter between two groups, with “**” indicated *p* < 0.01, “***” indicated *p* < 0.001, and “ns” indicated *p* > 0.05. Error bars showed ± SE (*n* ≥ 3).

**FIGURE 5:**
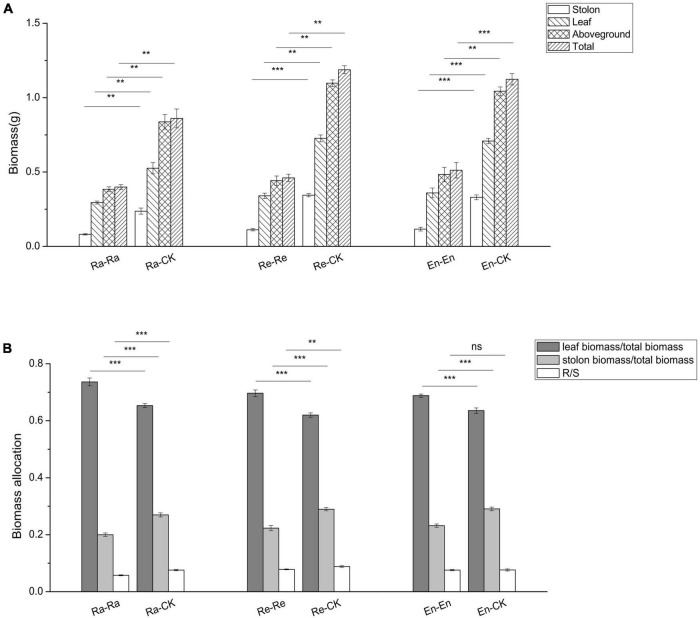
Biomass and its allocation of the clone in the second experiment stage under different offspring conditions. **(A)** Biomass; **(B)** biomass allocation. Different numbers of asterisks indicate significant differences of the same parameter between two groups, with “**” indicated *p* < 0.01, “***” indicated *p* < 0.001, and “ns” indicated *p* > 0.05. Error bars showed ± SE (*n* ≥ 3).

**FIGURE 6:**
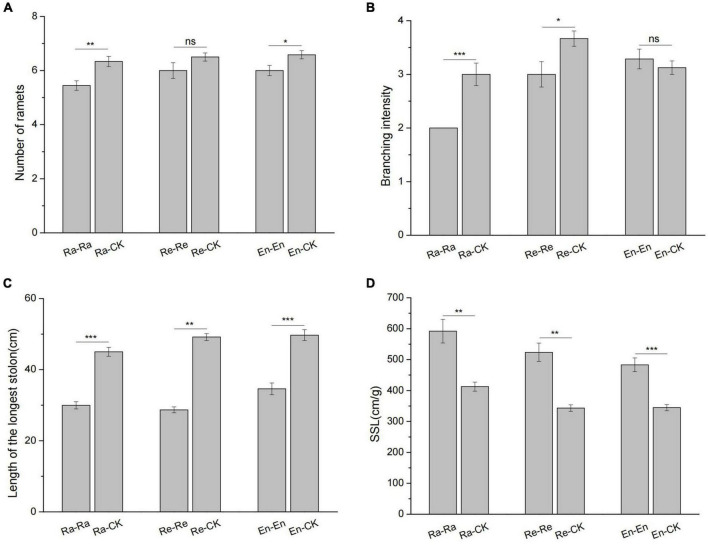
Growth architecture of clone in the second experiment stage under different offspring conditions. **(A)** The number of ramets; **(B)** branching intensity; **(C)** length of the longest stolon; **(D)** specific stolon length (SSL). Different numbers of asterisks indicate significant differences of the same parameter between two groups, with “*” indicated *p* < 0.05, “**” indicated *p* < 0.01, “***” indicated *p* < 0.001, and “ns” indicated *p* > 0.05. Error bars showed ± SE (*n* ≥ 3).

To study the effects of offspring current environment on offspring growth, the difference of offspring in ambient condition and UV-B environment was analyzed (Figures 2–6 and Table 6). When the offspring grew in the mismatched parental–offspring environments, lots of growth parameters, such as total biomass, aboveground and root biomass, root–shoot ratio, biomass of blade, leaf and petiole, blade area, and petiole length, were all increased significantly and the SPL was decreased, while the decreased SLA was only observed in Ra-CK (Figures 2, 3).

**TABLE 6:** Resource allocation of initial ramet in the second experiment stage under different offspring conditions.

Organ	Parameters	Ra-Ra	Ra-CK	Re-Re	Re-CK	En-En	En-CK
Blades	Total phosphorus (TP) (g/kg)	3.99 ± 0.09 b	7.54 ± 0.18 a	4.58 ± 0.15 b	8.01 ± 0.17 a	4.79 ± 0.13 b	7.24 ± 0.32 a
	Nitrate nitrogen (g/kg)	3.21 ± 0.05 a	2.69 ± 0.06 b	2.63 ± 0.06 a	2.17 ± 0.05 b	2.55 ± 0.10 a	2.09 ± 0.04 b
	Ammonium nitrogen (g/kg)	23.47 ± 0.15 b	28.61 ± 0.22 a	22.45 ± 0.11 b	28.50 ± 0.66 a	23.75 ± 0.49 b	27.81 ± 0.28 a
	Organic carbon (OC) (g/kg)	427.21 ± 2.15 a	399.64 ± 2.89 b	424.20 ± 0.60 a	402.90 ± 2.87 b	430.14 ± 1.56 a	406.81 ± 0.84 b
	Total nitrogen (TN) (g/kg)	26.68 ± 0.11 b	31.30 ± 0.17 a	25.08 ± 0.16 b	30.68 ± 0.61 a	26.31 ± 0.44 b	29.91 ± 0.29 a
	N:P	6.68 ± 0.17 a	4.16 ± 0.09 b	5.49 ± 0.20 a	3.83 ± 0.03 b	5.50 ± 0.06 a	4.15 ± 0.23 b
Roots	Total carbon (TC) (g/mg)	412.78 ± 4.46 b	439.54 ± 3.92 a	425.41 ± 1.36 a	424.34 ± 1.61 a	414.23 ± 3.10 a	427.78 ± 3.06 a
	Total nitrogen (TN) (g/kg)	19.55 ± 0.19 b	24.92 ± 0.20 a	19.25 ± 0.21 b	22.61 ± 0.17 a	21.39 ± 0.23 b	24.36 ± 0.08 a
	C:N	21.13 ± 0.41 a	17.64 ± 0.29 b	22.10 ± 0.29 a	18.77 ± 0.21 b	19.37 ± 0.07 a	17.56 ± 0.17 b

Values with different letters were significantly different between different offspring environments, whereas, the same letter indicates no significant differences (*P* < 0.05). Error bars showed ± SE (*n* ≥ 3). N:P, total nitrogen/total phosphorus (blades); C:N, total carbon/total nitrogen (roots).

There was no significant difference between the anthocyanin content of En-En and En-CK (0.049 vs. 0.035; *P* > 0.05) and between the flavonoid level of Ra-CK and Ra-Ra (1.680 vs. 1.781; *P* > 0.05), but flavonoid (1.930 vs. 1.335; *P* < 0.001) and anthocyanin (0.075 vs. 0.045; *P* < 0.01) level of Re-Re was higher than that of Re-CK, and the content of anthocyanin in Ra-CK was also higher than that of Ra-Ra (0.075 vs. 0.023; *P* < 0.001) (Figure 4).

The difference in TC of root was not significant in the predictable environments, but the TC of root decreased significantly in the Ra-Ra treatment by comparing with the Ra-CK (412.78 g/mg vs. 439.54 g/mg of Ra-CK; *P* < 0.05). There were significant differences in other indexes in the different offspring environments. TP, ammonium nitrogen, TN in blades, and TN in root decreased significantly after UV-B radiation, while nitrate-nitrogen, OC, N:P in blade, and C:N in root increased significantly under UV-B radiation (Table 6).

The biomass of all parts of the clone (biomass of stolon, leaf, and aboveground, and total biomass) decreased significantly after the offspring were exposed to the matched parental–offspring environments. Compared with offspring of the mismatched environment, the biomass allocation to leaf increased significantly, while the biomass allocation to stolon decreased significantly in the matched UV-B environments. The R/S decreased in the Ra-Ra (0.058 vs. 0.076 of Ra-CK; *P* < 0.001) and Re-Re (0.078 vs. 0.088 of Re-CK; *P* < 0.01), but there was no significant difference in the R/S between the En-CK and En-En (0.077 vs. 0.076; *P* > 0.05) (Figure 5).

In the Ra-Ra treatment, the number of ramets (5.4 vs. 6.3 of Ra-CK; *P* < 0.01) and branching intensity (2 vs. 3 of Ra-CK; *P* < 0.001) decreased significantly by comparing with the Ra-CK. There was no significant difference in the number of ramets between the Re-CK and Re-Re (6.5 vs. 6; *P* > 0.05). Compared with the Re-CK, the branching intensity decreased significantly in the Re-Re (3 vs. 3.67 of Re-CK; *P* < 0.05). In the En-En treatment, the number of ramets decreased significantly (6 vs. 6.6 of En-CK; *P* < 0.05), and the branching intensity had no significant change (3.29 vs. 3.13 of En-CK; *P* > 0.05) by comparing the En-CK. The longest stolons of the offspring became short and slender when they were exposed to UV-B radiation (Figure 6).

## Discussion

### The maternal effects on the growth strategy of clonal offspring

Maternal effects have become an important field of study in ecology, and there is an ongoing debate regarding their adaptive significance for offspring fitness (Marshall and Uller, 2007). Maternal effects can be either adaptive if they increase offspring fitness or not if they are neutral or harmful to the fitness of offspring (Galloway and Etterson, 2007; Donelson et al., 2018; Zhou et al., 2021). In this study, the clonal offspring of different UV-B radiated parents displayed various performances, despite they were in an unirradiated environment (Tables 2, 3). This difference of offspring was elicited by the maternal effects of parental environment. The maternal effects are regarded as highly contingent on the environmental variations. The environments with slowly and predictably changing select positive maternal effects, while, the environments with rapidly and unpredictably changing select negative maternal effects (Uller, 2008; Ezard et al., 2014). In our study, random UV-B radiation could be regarded as an unpredictable environment and regular and enhanced UV-B as two predictable environments. Obviously, diverse variations of parental environment triggered different maternal effects, which regulated different growth strategies of offspring. For offspring whose parent grow in unpredictable environments (random UV-B radiation), the content of defensive substances in blade was improved obviously to effectively reduce the damage of UV-B radiation to plant tissues. In addition, more carbon and nitrogen resources were allocated to roots. The offspring biomass was maintained (in clone) or even fully decreased (in initial ramets). Above all, rapid growth to increase biomass was not the goal of these offspring, and they invested more resources in enhancing defense process to resist the unpredictable UV-B stress. However, for the offspring of parents growing in predictable environment (regular and enhanced UV-B), their growth strategy was to combine both defense and growth processes. For instance, the contents of defensive compounds were also improved significantly, but the level was lower than that of offspring of unpredictable environmental parents. Moreover, the offspring growth was not influenced by the negative effects of parental UV-B. Of course, there were some growth differences between two kinds of offspring, such as the change of root biomass and resource allocation pattern. The offspring of regular radiated parent exhibited increased root biomass, but another offspring displayed regulated the allocation of nitrogen and phosphorus resources.

These diverse growth strategies induced by maternal UV-B effects were achieved by transgenerational plasticity, which had advantages in the corresponding environment. Transgenerational plasticity in response to maternal environments was common in plants (Galloway and Etterson, 2007). It could be found from these results that some traits showed high transgenerational plasticity, such as UV-B absorbing components, aboveground and underground biomass, petiole length, stolon length, and so on. While transgenerational plasticity was not observed in other traits (petiole biomass, blade area, ramets number, the level of N:P, ammonium nitrogen, and organic carbon).

In addition, the growth differences between predictable or unpredictable environments were attributed to both the changing intensity and frequency of UV-B environment experienced by the parental plants. In our study, the variation of environmental predictability contained the change of radiation frequency and/or intensity. The treatment of random UV-B radiation was to imitate the change of UV-B in nature, which included inconstant intensity and frequency of radiation. Meanwhile, only intensity changes were included in the enhanced UV-B radiation group. These variations among UV-B environments induced various effects and ultimately led to different growth performances.

### The influence of maternal effects on the adaptability of clonal offspring in ultraviolet-B environment

For the *G. longituba* in this study, when offspring grew in matched maternal–offspring UV-B environments, their growth was depressed. Among treatments, the ramets in the unpredictable environment as their mother ramets, the inhibition of growth was the strongest, while ramets in predictable environment accumulated more defensive (flavonoid and anthocyanin) components to resist UV-B radiation (Tables 2, 3). It seemed that maternal effects in unpredictable habitats were maladaptive, while some transgenerational effects of predictable environments were partially beneficial to improve the offspring fitness. Although some studies suggested that when the maternal environment is an accurate predictor of the environment that offspring will encounter, beneficial maternal effects are expected to promote adaptive shifts (Marshall and Uller, 2007; Ezard et al., 2014). However, whether the transgenerational effects in phenotype could be adaptive is difficult to interpret, as many of these fitness-linked traits responded in opposite directions, suggesting the potential for complex trade-offs among traits. The decrease in growth does not necessarily mean that maternal effect is completely unadaptable, and resources may be more devoted to production of chemical or physical defense. A trade-off between plant growth and defense to maintain optimal fitness has been reported in much research (Bazzaz et al., 1987; Züst and Agrawal, 2017; Guo et al., 2018; Dwivedi et al., 2021). According to the acclimatory response hypothesis, activating defenses is an adaptive response. Plants will reconfigure their metabolic and allocation strategies to optimize the use of potentially limiting resources (Ballaré and Austin, 2019). Another hypothesis for the growth-defense trade-off is that growth, at least in some ways, is not suitable in certain stresses (Díaz et al., 2001; Ballaré and Austin, 2019). As such, it was beneficial for the ramets of *G. longituba* to reduce growth to minimize exposure to UV-B radiation. They put more energy into chemical defense in predictable UV-B environments, which is an economically feasible strategy. It is pointed out that plants will avoid excessive growth or defense through a negative feedback-regulatory loop and achieve balance in response to adverse environments (Li et al., 2019). In brief, predictable maternal conditions experienced by clonal *G. longituba* can affect growth of offspring, and some phenotypes induced by the transgenerational effects may be adaptive. These responses ultimately allow offspring to tolerate the stress conditions they currently experienced.

### The influence of offspring environment on their performance

In our study, if UV-B radiation was released, the growth of offspring became better, and it could be observed from the recovery of all organ biomass, which was most significant in offspring of predictable radiated parent (Figures 2, 5). The performance of offspring was related to the within-generation plasticity. Within-generation plasticity relies on the current environmental information (Auge et al., 2017a). This compensation of growth under no UV-B condition was attributed to the recovery of leaf, the enlargement of blade area contributed to absorbing more light for photosynthesis, and more photosynthate transferred to roots, helping to increase the root biomass. However, the maternal effects still played the role in offspring growth, and it could be found from the increased flavonoid content. The defenses induced in parents can be inherited by offspring, allowing progenies to deploy stronger defenses had been discovered (Karasov et al., 2017). It had been suggested that the growth and behavior of clonal plants might be significantly modified by environments that the parent had experienced but are no longer present, and the methylation variations of offspring inherited from the parents make the effects on their phenotype (Chinnusamy and Zhu, 2009; Verhoeven et al., 2010; Huber et al., 2014). Of course, the maternal effects might reduce, and the limitation of transgenerational transmission in response to stress has also been suggested in some studies, which may also contribute to traits recovery (Boyko et al., 2010; Zhang et al., 2021). In short, in our study, the performance of clonal offspring was affected by the combination of within- and transgenerational plasticity.

## Conclusion

In our study, maternal effects play an important role in shaping the growth strategies of clonal offspring, and the effects were regulated by predictability of parental environment. Maternal effects induced by unpredictable environment affected offspring growth by investing more resources in defense than rapid growth, while that by predictable environment regulated the growth of offspring via combining the two processes of defense and growth. However, there were still some differences between the offspring of regular and enhanced radiated parents. In addition, when the offspring were exposed to the matched parental–offspring UV-B environments, the adaptability of maternal effects varied with different predictability cues. It seemed that transgenerational effects of predictable environments were beneficial in improving the adaption of offspring partly. Clonal offspring in predictable habitats devoted more resources to chemical defense while also trying to maintain growth. Besides, when the offspring were transplanted to the non-UV-B environment, their growth was significantly recovered for the within-generational and transgenerational plasticity. Our findings suggest that environmental predictability plays an important role in the trade-off between plant growth and defense.

## Data availability statement

The data presented in the study are included in the article, further inquiries can be directed to the corresponding author.

## Author contributions

XL conceived and designed the experiments. YG, JQ, and XW performed the experiments. YG wrote the manuscript. JQ, XW, ZZ, XL, RZ, and MY provided data analysis and editorial advice. All authors contributed to the article and approved the submitted version.
